# The APC/C E3 ligase subunit ANAPC11 mediates FOXO3 protein degradation to promote cell proliferation and lymph node metastasis in urothelial bladder cancer

**DOI:** 10.1038/s41419-023-06000-x

**Published:** 2023-08-12

**Authors:** Dong Yan, Qingqing He, Lu Pei, Meihua Yang, Lifang Huang, Jianqiu Kong, Wang He, Hao Liu, Shizhong Xu, Haide Qin, Tianxin Lin, Jian Huang

**Affiliations:** 1 Department of Urology, Sun Yat-sen Memorial Hospital, Sun Yat-sen University, Guangzhou, China; 2 Guangdong Provincial Key Laboratory of Malignant Tumor Epigenetics and Gene Regulation, Sun Yat-sen Memorial Hospital, Sun Yat-sen University, Guangzhou, China; 3 State Key Laboratory of Oncology in South China, Collaborative Innovation Center for Cancer Medicine, Guangdong Key Laboratory of Nasopharyngeal Carcinoma Diagnosis and Therapy, Sun Yat-sen University Cancer Center, Guangzhou, China; 4 Department of Urology, The First Affiliated Hospital of Nanchang University, Nanchang, China

**Keywords:** Bladder cancer, Cell invasion

## Abstract

Urothelial bladder cancer (UBC) is one of the most prevalent malignancies worldwide, with striking tumor heterogeneity. Elucidating the molecular mechanisms that can be exploited for the treatment of aggressive UBC is a particularly relevant goal. Protein ubiquitination is a critical post-translational modification (PTM) that mediates the degradation of target protein via the proteasome. However, the roles of aberrant protein ubiquitination in UBC development and the underlying mechanisms by which it drives tumor progression remain unclear. In this study, taking advantage of clustered regularly interspaced short palindromic repeats (CRISPR)-CRISPR-associated protein (Cas) 9 technology, we identified the ubiquitin E3 ligase ANAPC11, a critical subunit of the anaphase-promoting complex/cyclosome (APC/C), as a potential oncogenic molecule in UBC cells. Our clinical analysis showed that elevated expression of ANAPC11 was significantly correlated with high T stage, positive lymph node (LN) metastasis, and poor outcomes in UBC patients. By employing a series of in vitro experiments, we demonstrated that ANAPC11 enhanced the proliferation and invasiveness of UBC cells, while knockout of ANAPC11 inhibited the growth and LN metastasis of UBC cells in vivo. By conducting immunoprecipitation coupled with mass spectrometry, we confirmed that ANAPC11 increased the ubiquitination level of the Forkhead transcription factor FOXO3. The resulting decrease in FOXO3 protein stability led to the downregulation of the cell cycle regulator p21 and decreased expression of GULP1, a downstream effector of androgen receptor signaling. Taken together, these findings indicated that ANAPC11 plays an oncogenic role in UBC by modulating FOXO3 protein degradation. The ANAPC11–FOXO3 regulatory axis might serve as a novel therapeutic target for UBC.

## Introduction

Urothelial bladder cancer (UBC) is the tenth most prevalent cancer worldwide and is responsible for nearly 170,000 deaths per year [1, 2]. Approximately 75% of UBC cases are classified as non-muscle-invasive bladder cancer (NMIBC) at initial diagnosis [3]. However, 15% of NMIBCs will eventually progress to high-risk muscle-invasive bladder cancer (MIBC) [4]. Moreover, specific molecular subtypes, such as the luminal infiltrated subtype or basal-squamous subtype with stromal enrichment, exhibit aggressive behavior, regardless of the histological stage [5, 6]. To date, the molecular mechanisms for UBC development and progression are largely unknown.

Ubiquitination is a multifunctional and reversible post-translational modification (PTM) that potentially increases the diversity of protein functional profiles [7, 8]. Ubiquitin (Ub), a 76-amino acid protein, contains one N-terminal methionine residue (M1) and seven lysine residues (K6, K11, K27, K29, K33, K48, K63) that function as linkage sites for various polyubiquitin chain modifications to determine the outcomes of the target proteins, which include proteasome-dependent degradation, autophagy and signal transduction [9–11]. Monoubiquitination is related to DNA damage repair [12]. Covalent ubiquitination is achieved by a three-step cascade involving E1 (activating enzyme), E2 (conjugating enzyme), and E3 (ligase) [13]. Moreover, Ub can be removed from the substrate protein by deubiquitinating enzymes (DUBs) [14, 15]. Previous studies have shown that aberrant ubiquitination activity is involved in the pathogenesis of numerous cancers [16, 17]. Ubiquitination plays important roles in cell cycle regulation, cytokine signaling, epithelial-mesenchymal transition, and innate immunity [18–20]. Studies on ubiquitination-mediated protein degradation have led to the development of novel therapeutics, such as proteolysis-targeting chimeras (PROTACs), which can degrade a wide range of proteins of interest by facilitating their polyubiquitination and subsequent proteasome-mediated proteolysis [21, 22]. Therefore, elucidating the mechanisms of ubiquitination in driving UBC progression is a particularly relevant goal.

Clustered regularly interspaced short palindromic repeats (CRISPR) and CRISPR-associated protein (Cas) were first identified in bacteria as constitutes of the adaptive immune system targeting specific foreign DNA molecules [23, 24]. The CRISPR system has been engineered to be a powerful and versatile tool for genome editing and epigenetic modification [25–27]. CRISPR–Cas9 screens efficiently aid in identifying druggable targets, drug-resistant mutations, and synthetic lethality due to their accurate base-base recognition mechanism [28–30]. In this study, we surveyed CRISPR–Cas9 datasets from DepMap and identified the APC/C E3 ligase subunit ANAPC11 as an essential gene in UBC cells [31]. We confirmed that knockdown of ANAPC11 inhibited UBC cell proliferation and migration, while overexpression of ANAPC11 promoted aggressive phenotypes in UBC cells. Mechanistically, we found that ANAPC11 elevated the ubiquitination of Forkhead transcription factor FOXO3 to decrease its stability, mediating the downregulation of the cell cycle regulator p21 and a novel lipid metabolism molecule GULP1. Thus, in the present study, we elucidated the mechanisms by which ANAPC11 governs FOXO3 protein degradation to promote urothelial carcinoma cell proliferation and LN metastasis. The ANAPC11–FOXO3 axis might serve as a potential therapeutic target for UBC.

## Materials and methods

### Cell culture

The human bladder urothelial carcinoma cell lines HT-1376, UM-UC-3, T24, J82, 5637, and RT4, the human immortalized uroepithelial cell line SV-HUC-1 and the human embryonic kidney cell line 293 T were obtained from the American Type Culture Collection. EJ cells were purchased from the Institute of Biochemistry of the Chinese Academy of Science. Cells were cultured in different media (EMEM for HT-1376 and J82; DMEM for UM-UC-3, 293 T, and EJ; RPMI-1640 for T24 and 5637; McCoy’s 5A for RT4; F12K for SV-HUC-1) supplemented with 10% fetal bovine serum (ExCell bio, China) and 1% penicillin/streptomycin (Thermo Fisher, USA) at 37 °C with 5% of CO_2_.

### Western blot analysis

Cells were lysed with RIPA buffer (Beyotime, China) supplemented with 1% protease/phosphatase inhibitors (CWBIO, China), and the protein concentration was calculated using a Pierce^TM^ BCA Protein Assay Kit (Thermo Fisher, USA). Equal amounts of samples were loaded into 10% SDS‒PAGE gels, and separated proteins were then transferred onto PVDF Western Blotting Membranes (Roche, Switzerland). The membranes were incubated with specific primary antibodies overnight after blocking with 5% skim milk powder for 1 h. The primary antibodies used were anti-ANAPC11 (14090, CST, USA), anti-GAPDH (AC001, Abclonal, China), anti-FOXO3 (NBP2-16521, Novus Biologicals, USA), anti-His (RM1001, Beijing Ray Antibody Biotech, China), anti-HA (51064-2-AP, Proteintech, China), anti-ubiquitin (YM3636, ImmunoWay, USA), anti-p21 (2947, CST, USA) and anti-GULP1 (19902-1-AP, Proteintech, China). The next day, membranes were incubated with HRP-conjugated secondary antibodies (Goat Anti-Rabbit IgG and Goat Anti-Mouse IgG, CWBIO, China), and immunoreactions were visualized and imaged by a SmartChemi^TM^ system (SAGE, China). Band intensities were quantified via ImageJ software.

### Patients and samples

A cohort of 110 UBC patients who underwent surgery at Sun Yat-sen Memorial Hospital, Sun Yat-sen University, was included in this study. Informed consent was obtained from each patient. The pathological diagnosis of all patients was confirmed, and the clinicopathological characteristics of the patients are summarized in Table 1. This study was approved by the Ethics Committees of Sun Yat-sen Memorial Hospital, Sun Yat-sen University.

**Table 1:** Correlation of ANAPC11 expression and clinical parameters.

Characteristics	ANAPC11 expression				
	No. (%)	Low (%)	High (%)		*P*-value
*Age*					
<60	35 (31.8)	19 (54.3)		16 (45.7)	0.051
≥60	75 (68.2)	26 (34.7)		49 (65.3)	
*Sex*					
Male	97 (88.2)	40 (41.2)		57 (58.8)	0.848
Female	13 (11.8)	5 (38.5)		8 (61.5)	
*T stage*					
Ta-T1	47 (42.7)	27 (57.4)		20 (42.6)	0.002**
T2-T4	63 (57.3)	18 (28.6)		45 (71.4)	
*LN status*					
LN−	95 (86.4)	44 (46.3)		51 (53.7)	0.004**
LN+	15 (13.6)	1 (6.7)		14 (93.3)	
*Grade*					
Low	18 (16.4)	16 (88.9)		2 (11.1)	0.000**
High	92 (83.6)	29 (31.5)		63 (68.5)	
Total	110	45		65	

Chi-square test.

**P < 0.01.

### Immunohistochemical (IHC) staining

Paraffin-embedded tissues were rehydrated and subjected to antigen retrieval before incubation with anti-ANAPC11 (NBP1-78050, Novus Biologicals, USA), anti-FOXO3 (NBP2-16521, Novus Biologicals, USA), anti-p21 (2947, CST, USA), anti-GULP1 (19902-1-AP, Proteintech, China) or anti-Ki67 (GB111499, Servicebio, China) antibodies at 4 °C overnight. After incubation with horseradish peroxidase-conjugated secondary antibodies, the specimens were stained with diaminobenzidine (DAB) and hematoxylin. The abundance of ANAPC11 in each specimen was evaluated by determining the *H*-score as previously described [32, 33]. The intensity of staining was categorized as follows: no staining (0), weak staining (1), intermediate staining (2), and strong staining (3). The *H*-score was calculated as Σ(intensity × percentage of tumor cells) and ranged from 0 to 300.

### Transfection, cycloheximide (CHX), and MG132 assays

For transient transfection, small interfering RNAs (siRNAs) (GenePharma, China) were synthesized and transfected via Lipofectamine RNAiMAX (Invitrogen, USA). The open reading frame (ORF) of ANAPC11, FOXO3 (His-tagged), or an NC sequence was inserted into the pcDNA3.1 plasmid, and transfection of plasmids was performed with X-tremeGENE (Sigma, USA). For stable transfection, the plasmid containing a short hairpin RNA (shRNA) sequence specifically targeting ANAPC11 was constructed using the pLVX-shRNA2-Puro backbone. The plasmid used for CRISPR‒Cas9-mediated knockout (KO) of ANAPC11 was produced following Zhang’s protocol using the lentiCRISPR v2 backbone [25]. The shRNA or CRISPR vector was transfected into 293 T cells along with the psPAX2 and pMD2.G plasmids for viral packaging. Cells were infected with lentivirus and selected with puromycin. The sequences of the siRNAs, shRNA, and single guide RNA (sgRNA) are listed in Supplementary Table 1.

CHX or MG132 was added to the medium to a final concentration of 30 μg/mL or 20 μM, respectively. Cells were collected at the indicated time points for CHX assays or 48 h after MG132 treatment.

### 3-(4,5-Dimethylthiazol-2-yl)-5-(3-carboxymethoxyphenyl)-2-(4-sulfophenyl)-2H-tetrazolium (MTS) assay, colony formation assay, flow cytometric analyses of the cell cycle and apoptosis, wound healing assay and Transwell migration and invasion assays

MTS assays, colony formation assays and flow cytometry, wound healing assays, and Transwell migration and invasion assays were conducted as previously described [34].

### Immunoprecipitation (IP) and mass spectrometry

UBC cells subjected to different treatments were collected and lysed with cell lysis buffer for WB and IP (APE × BIO, USA). After centrifugation, the supernatant was quantified, and equal amounts of protein were incubated with Protein A/G magnetic beads (Biolinkedin, China) conjugated with specific antibodies at 4 °C overnight. The beads were washed, and the precipitated proteins were eluted and subjected to western blotting and mass spectrometry. Mass spectrometry was performed by the Bioinformatics and Omics Center, Sun Yat-Sen Memorial Hospital, Sun Yat-Sen University.

### Glutathione S-transferase (GST) pulldown assay

The coding sequence of ANAPC11 was inserted into the pGEX-6P-3 vector (IGEbio, China). The resulting plasmid was transformed into *E. coli* and the expression of the GST-ANAPC11 fusion protein was induced by treatment with 1 mM IPTG. To purify the fusion protein, glutathione Sepharose beads (Abcam, UK) were used according to the manufacturer’s instructions. The fusion protein was further incubated with T24 or UM-UC-3 cell lysates, and the eluted complexes were detected by Coomassie blue staining and western blotting analysis.

### RNA isolation, reverse transcription, real-time PCR (RT-PCR), and chromatin immunoprecipitation (ChIP)

RNA was extracted using an RNA-Quick Purification Kit (ES Science, China). Thereafter, 1 μg of total RNA was reverse transcribed with PrimeScript RT Master Mix (Takara, Japan) following the manufacturer’s instructions. RT-PCR was performed with Hieff UNICON^®^ Power qPCR SYBR Green Master Mix (YEASEN, China) on a QuantStudio Dx instrument (Applied Biosystem, USA). ChIP was conducted using a Pierce Magnetic ChIP Kit (Thermo Fisher, USA). Briefly, cells were subjected to 1% formaldehyde treatment for cross-linking. Then, the cells were lysed, and the lysates were subjected to MNase digestion and sonication to obtain DNA fragments. After immunoprecipitation with an anti-FOXO3 antibody (NBP2-16521, Novus Biologicals, USA), samples were treated with Proteinase K and recovered with a DNA Clean-Up Column. The primers used are listed in Supplementary Table 2.

### Dual-luciferase reporter assay

The promoter (−2000 bp to −1 bp upstream of the transcription start site (TSS)) sequences of *CDKN1A* and *GULP1* were inserted into the pGL3-basic vector separately. Then, pGL3-basic, pRL-TK, and pcDNA3.1 with an NC sequence or the FOXO3 ORF were co-transfected into 293 T cells. Forty-eight hours after transfection, firefly, and *Renilla* luminescence were measured using the Dual-Glo^®^ Luciferase Assay System (Promega, USA). Relative firefly luminescence was normalized to *Renilla* luminescence.

### Animal experiments

Four-week-old male BALB/c nude mice were randomly divided into control and experimental groups (*n* = 5 mice for each group). For the subcutaneous xenograft model, 5 × 10^6^ ANAPC11-NC or ANAPC11-KO T24 cells in 100 μl PBS were injected into the left flank of each mouse, and the tumor volume was calculated every 5 days with the following equation: volume (mm^3^) = 1/2 × (length) × (width)^2^. Mice were euthanized 30 days after cell injection, and tumor sections were fixed, embedded, and subjected to hematoxylin and eosin (HE), and IHC staining. For the LN metastasis model, control and ANAPC11 KO cells were infected with Lenti-luciferase P2A-Neo and selected with G-418 (1 mg/ml). T24 cells (5 × 10^6^) in 50 μl PBS were injected into the right footpads of the mice. The mice were intraperitoneally injected with luciferin and anesthetized with 3% isoflurane 10 min later. Bioluminescence was detected and imaged with an AniView 600 system (BLT, China). The procedures for the animal experiments were evaluated and approved by the Institutional Animal Care and Use Committee of Sun Yat-sen University in compliance with the Guide for the *Care and Use of Laboratory Animals*.

### Statistical analysis

Data from at least three independent experiments were analyzed by SPSS 20.0 (IBM SPSS Statistics, USA) and are presented as the mean ± standard deviation (SD) values. Student’s *t*-test, the chi-square test, the Mann‒Whitney *U* test, and one-way analysis of variance (ANOVA) were applied to compare differences among groups based on the data type. The log-rank test was conducted after Kaplan–Meier survival analysis to assess patient’s prognosis. *P* < 0.05 was considered statistically significant.

## Results

### ANAPC11 overexpressed is associated with poor prognosis in UBC

**Fig. 1:**
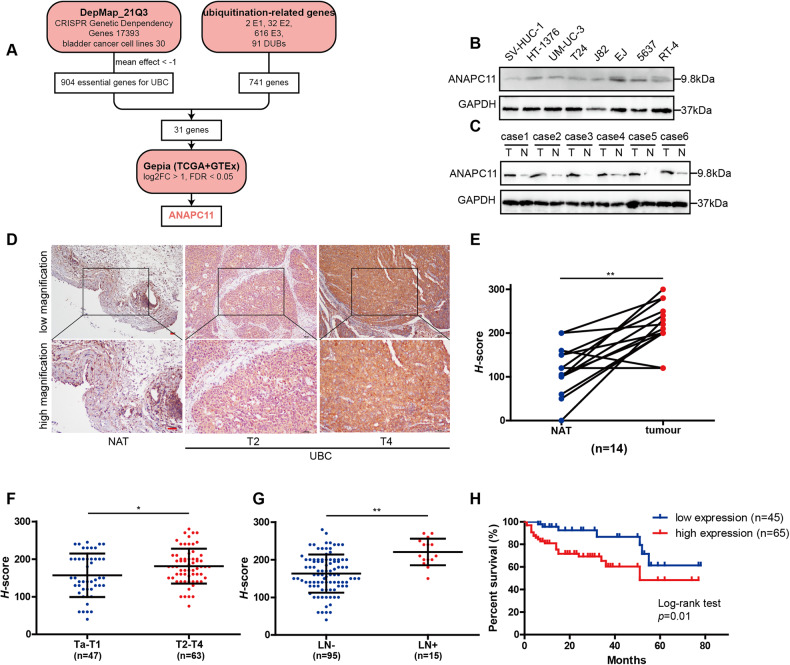
ANAPC11 overexpression is associated with poor prognosis in UBC. **A** Flow diagram showed a brief illustration of how ANAPC11 was screened out. **B** Western blot analysis of the expression of ANAPC11 in immortalized uroepithelial cell line (SV-HUC-1) and seven UBC cell lines (HT-1376, UM-UC-3, T24, J82, E-J, 5637, and RT-4). **C** Western blot analysis of ANAPC11 expression in six UBC patient specimens. T for tumor and N for NAT. **D** Representative images of ANAPC11 expression in the NAT and UBC with different T stages, evaluated by IHC staining. **E** *H*-scores of 14 paired UBC and NAT specimens. Paired, two-tailed, Mann-Whitney *U* test. **F**, **G** *H*-scores of 110 UBC patients’ tissues compared by T stage and LN status. Mann–Whitney *U* test. **H** The Kaplan–Meier survival curve is comparing 110 patients’ survival with relatively low or high expression of ANAPC11. Log-rank test. Data are shown as mean ± SD. ^*^P < 0.05, ^**^P < 0.01. Scale bar = 100 μm.

To identify genetic dependencies and the biomarkers for UBC, we used the DepMap 21Q3 dataset containing the results of a genome-wide CRISPR‒Cas9 screen of a total of 30 bladder cancer cell lines, and 904 genes were selected by the following criterion: mean effect < −1. Then, we defined a subset of 741 ubiquitination-related genes, including 2 E1 [35], 32 E2 [36], 616 E3 [37], and 91 DUB [38], according to previous studies. The subset of ubiquitination-related genes identified in this study is summarized in Supplementary Table 3. After cross-comparing these two subsets, we found 31 overlapping genes. We validated all 31 genes in the Gepia database (including TCGA and GTEx data, log2FC > 1, FDR < 0.05), and only ANAPC11 was screened out and considered to have potential ubiquitination-related dependencies in UBC (Fig. 1A). ANAPC11 has been documented as the critical component of APC/C, a multifunctional E3 ligase regulating various cellular processes. Due to the important roles of the ubiquitination machinery in neoplasms and its potential value for clinical translation, in combination with the specific aim of understanding the roles of APC/C proteins in UBC, we further comprehensively characterized ANAPC11 to investigate its biological functions and its underlying mechanisms in driving UBC progression.

Next, the expression of ANAPC11 in seven commonly used UBC cell lines and the human immortalized uroepithelial cell line SV-HUC-1 was measured via western blotting, and we found that ANAPC11 had higher expression in the UBC cell lines (Fig. 1B). We then validated the abundance of ANAPC11 in UBC patients. In six UBC specimens, ANAPC11 was consistently upregulated compared with its expression in normal adjacent tissues (NATs), as determined by western blotting (Fig. 1C). We further evaluated the expression of ANAPC11 via IHC staining (Fig. 1D). In 14 paired paraffin-embedded UBC and NAT sections, ANAPC11 had higher *H*-scores in UBC tissues than in NATs (Fig. 1E). In the cohort of 110 UBC patients, high expression of ANAPC11 was associated with higher T stage, positive lymph node (LN) metastasis, and higher grade but not with age or sex (Fig. 1F, G, Table 1). Moreover, high expression of ANAPC11 indicated worse overall survival in UBC patients (Fig. 1H). These results suggest that ANAPC11 may be of vital importance in UBC progression.

### ANAPC11 promotes the proliferation and migration of UBC cells in vitro

**Fig. 2:**
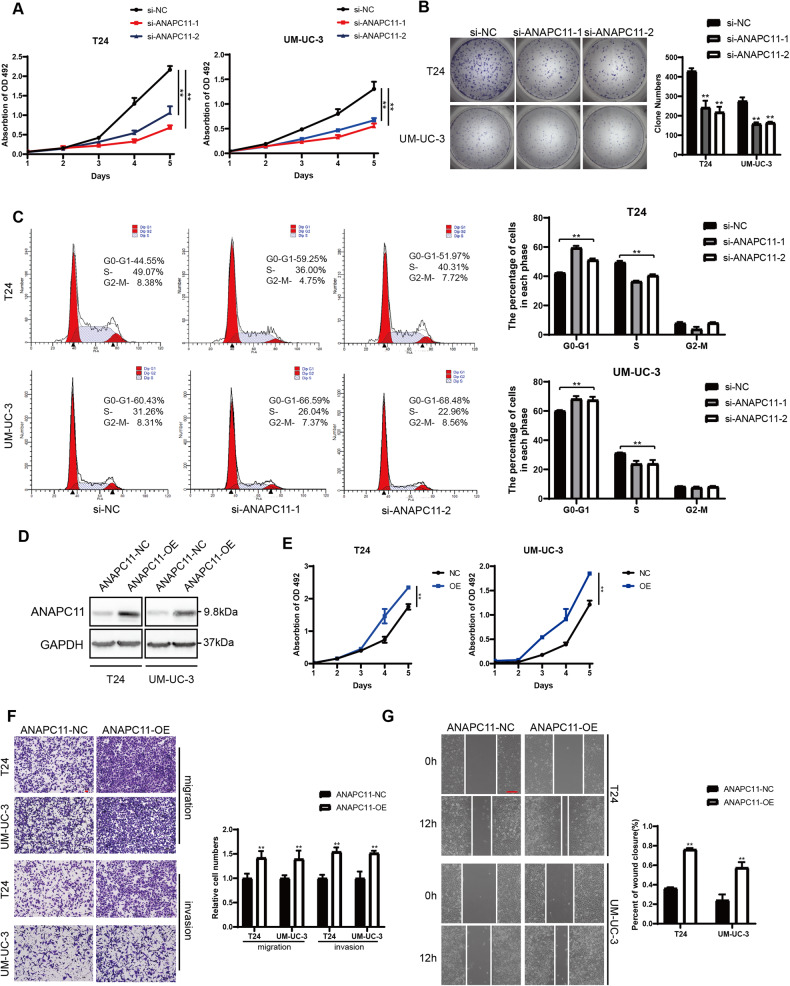
ANAPC11 promotes the proliferation and migration of UBC cells in vitro. **A** MTS assays measured the growth rates of UBC cells knocking down ANAPC11. ANOVA. **B** Representative images and histogram analysis of colony formation assays with T24 and UM-UC-3 cells treated with indicated siRNAs. Unpaired, two-tailed student’s *t*-test. **C** Representative images and quantification of cells in each phase within the cell cycle measured by flow cytometry. **D** The expression of ANAPC11 in UBC cells transfected with indicated plasmid measured by western blotting. **E** MTS assays of cells overexpressing ANAPC11. ANOVA. **F** Cell migratory and invasive abilities of UBC cells transfected with ANAPC11-NC or ANAPC11-OE plasmid were evaluated by Transwell migration and invasion assays. Unpaired, two-tailed student’s *t*-test. Scale bar = 100 μm. (**G**) Wound healing assays showed the migratory abilities of UBC cells. Unpaired, two-tailed student’s *t-*test. Scale bar = 200 μm. Data are shown as mean ± SD. ^**^P < 0.01.

To investigate the biological roles of ANAPC11 in UBC, T24, and UM-UC-3 cells were selected for further study, we transfected siRNAs into T24 and UM-UC-3 cells to transiently knock down ANAPC11. Western blot analysis proved the knockdown efficiency of the siRNAs (Supplementary Fig. 1A). MTS assays were conducted to evaluate the viability of UBC cells, and the knockdown of ANAPC11 was found to reduce the growth rate of UBC cells (Fig. 2A). We also found that silencing ANAPC11 robustly reduced the colony formation capacity of UBC cells (Fig. 2B). To quantify the effect of ANAPC11 on the cell cycle and apoptosis, flow cytometry was performed, and silencing ANAPC11 was found to increase the proportion of cells in G0/G1 phase, while the percentage of cells in S phase was reduced (Fig. 2C). However, knockdown of ANAPC11 did not significantly alter the apoptosis of UBC cells (Supplementary Fig. 1B). We conducted Transwell migration, Transwell invasion, and wound healing assays and found that ANAPC11 knockdown decreased the migration and invasion abilities of UBC cells (Supplementary Fig. 1C-D). Then we transfected the pcDNA3.1 plasmid containing the ANAPC11 ORF into UBC cells to overexpress ANAPC11 (Fig. 2D). As expected, elevated expression of ANAPC11 increased the growth rate, colony formation ability, and the migration, invasion and wound healing capacities of UBC cells (Fig. 2E–G; Supplementary Fig. 1E). These results indicate that ANAPC11 enhances the proliferation and invasiveness of UBC cells in vitro.

### ANAPC11 mediates FOXO3 degradation through the ubiquitin–proteasome pathway

**Fig. 3:**
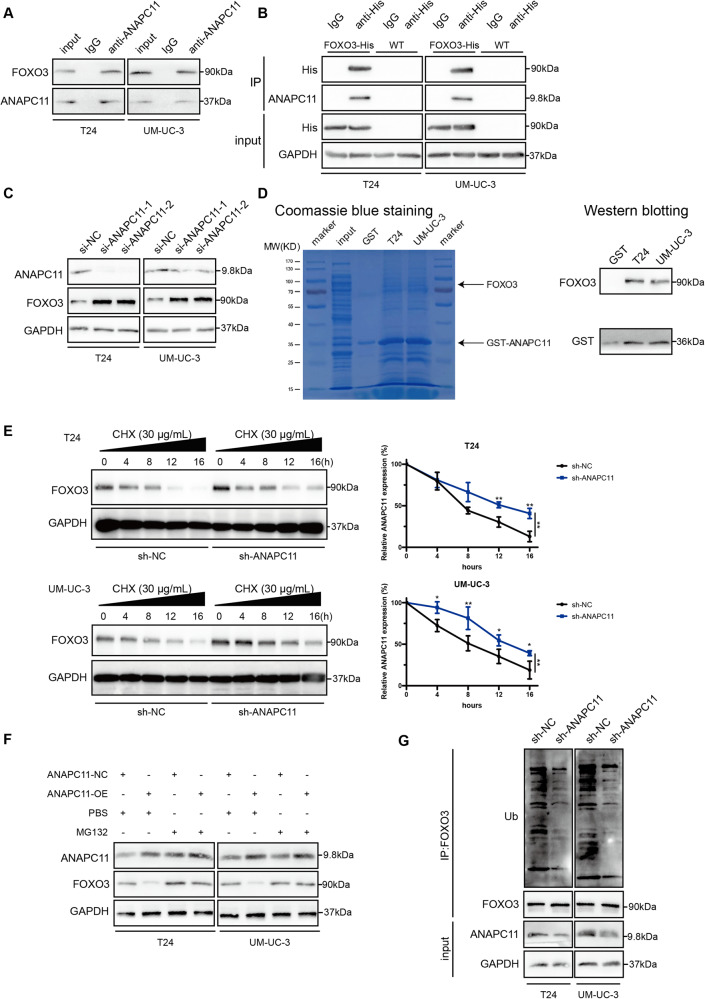
ANAPC11 mediates FOXO3 degradation through the ubiquitin–proteasome pathway. **A** Western blot analysis followed by IP in T24 and UM-UC-3 cells with IgG or anti-ANAPC11 antibody. **B** IP with anti-His or IgG in T24 and UM-UC-3 cells transfected with FOXO3-His or not. **C** Protein levels of ANAPC11, FOXO3, and GAPDH were measured by western blot analysis. **D** GST pulldown demonstrated by Coomassie blue staining (left) and western blotting (right). **E** Abundance of FOXO3 in sh-NC or sh-ANAPC11 UBC cells treatment with 30 μg/mL CHX, detected by western blotting. Band intensities were quantified by ImageJ and normalized to the intensity of 0 h. **F** Western blot analysis of cells overexpressing ANAPC11 treated with PBS or MG132 (20 μM). **G** Western blot analysis followed by IP evaluated the ubiquitination of FOXO3. Data are shown as mean ± SD. ANOVA, ^*^P < 0.05, ^**^P < 0.01.

Previous studies have established that ANAPC11 is the catalytic core subunit of APC/C and functions as an E3 ubiquitin ligase [39]. We investigated whether ANAPC11 regulates UBC cell growth and metastasis through the ubiquitin‒proteasome system (UPS). We performed IP of ANAPC11 followed by mass spectrometry in UM-UC-3 cells to identify molecules interacting with ANAPC11 (Supplementary Fig. 2A) and identified a series of proteins, including known APC/C components and cofactors such as ANAPC1, ANAPC2, ANAPC4, ANAPC5, ANAPC7, ANAPC10, CDC16, CDC23, and CDC27, which indirectly proved the specificity of the IP assays [40]. Among these factors, we selected FOXO3 for further investigation for the following reasons: (i) FOXO3 is an evolutionarily conserved transcription factor that plays a vital role in carcinogenesis, cell cycle regulation, apoptosis, and DNA damage repair [41–43]; (ii) we repeated the IP experiments in both T24 and UM-UC-3 cells and successfully validated the interaction between ANAPC11 and FOXO3 via western blot analysis (Fig. 3A); (iii) His-labeled FOXO3 was transfected into UBC cells, and ANAPC11 was enriched by IP with an anti-His antibody (Fig. 3B); and (iv) knocking down ANAPC11 elevated but overexpression of ANAPC11 reduced the protein level of FOXO3 (Fig. 3C; Supplementary Fig. 2B). Since ANAPC11 is a catalytic subunit of APC/C, GST pulldown was conducted to rule out indirect binding, and it was proven that FOXO3 physically interacts with ANAPC11 (Fig. 3D).

To clarify how the FOXO3 abundance is modulated by ANAPC11, we silenced ANAPC11 with siRNAs in T24 and UM-UC-3 cells, and RT-PCR analysis revealed that the FOXO3 mRNA level was not significantly and consistently altered, suggesting that ANAPC11 may post-transcriptionally regulate FOXO3 (Supplementary Fig. 2C). UBC cells with stable silencing of ANAPC11 were constructed via lentiviral transduction of sh-NC or sh-ANAPC11 and the silencing efficiency was confirmed by western blot analysis (Supplementary Fig. 2D). We used CHX to halt transcription in UBC cells and collected cells at the indicated times to analyze whether ANAPC11 affected the stability of FOXO3. Silencing of ANAPC11 in UBC cells resulted in a prolonged half-life of FOXO3 (Fig. 3E). Moreover, treatment with the proteasome inhibitor MG132 abolished the effect of ANAPC11 on FOXO3 stability, suggesting that ANAPC11 degraded FOXO3 through the ubiquitin‒proteasome pathway (Fig. 3F). Furthermore, the ubiquitination state of FOXO3 in UBC cells was determined via IP, and silencing of ANAPC11 was found to significantly decrease the ubiquitination of FOXO3 (Fig. 3G). It has been reported that K11- and K48-linked polyubiquitination results in 26S proteasome-mediated proteolysis [9]. Therefore, we transfected K11- and K48-linked Ub into UBC cells separately and observed an increase in K11-linked polyubiquitination but not K48-linked polyubiquitination in UBC cells overexpressing ANAPC11 (Supplementary Fig. 2E-F). Taken together, the evidence above showed that ANAPC11 acted as an E3 Ub ligase to mark FOXO3 for degradation through the UPS.

### FOXO3 is a critical tumor suppressor in UBC

**Fig. 4:**
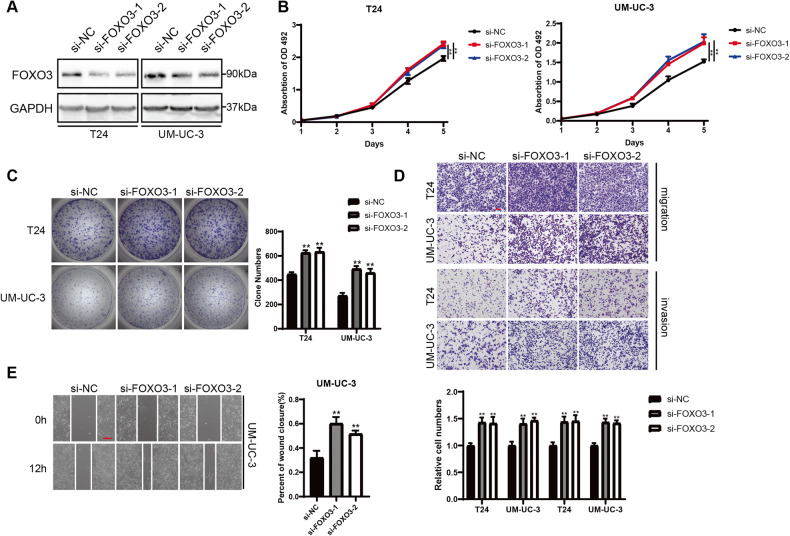
FOXO3 is a critical tumor suppressor in UBC. **A** Western blot analysis showed the abundance of FOXO3 in T24 and UM-UC-3 cells treated with indicated siRNAs. **B** Cell proliferation of UBC cells silencing FOXO3, shown by MTS assays. ANOVA. **C** Representative images and histogram analysis of colony formation assays with T24 and UM-UC-3 cells treated with indicated siRNAs. Unpaired, two-tailed student’s *t*-test. **D** Representative images and relative cell courts of Transwell migration and invasion assays. Unpaired, two-tailed student’s *t*-test. **E** Images of wound closure were captured, and the percentage of wound closure was demonstrated. Unpaired, two-tailed student’s *t*-test. Data are shown as mean ± SD. ^**^P < 0.01.

Then, we designed and synthesized specific siRNAs targeting FOXO3 mRNA to downregulate FOXO3 in UBC cells (Fig. 4A). In accordance with the effects of ANAPC11 overexpression, silencing of FOXO3 robustly increased the viability and colony formation ability of T24 and UM-UC-3 cells (Fig. 4B, C). Decreased FOXO3 levels also resulted in enhanced migration and invasion abilities in UBC cells compared with negative control cells (Fig. 4D, E). Collectively, these data indicated that FOXO3 played a tumor-suppressive role in UBC.

### Overexpression of FOXO3 abrogates ANAPC11-mediated progression in UBC cells

**Fig. 5:**
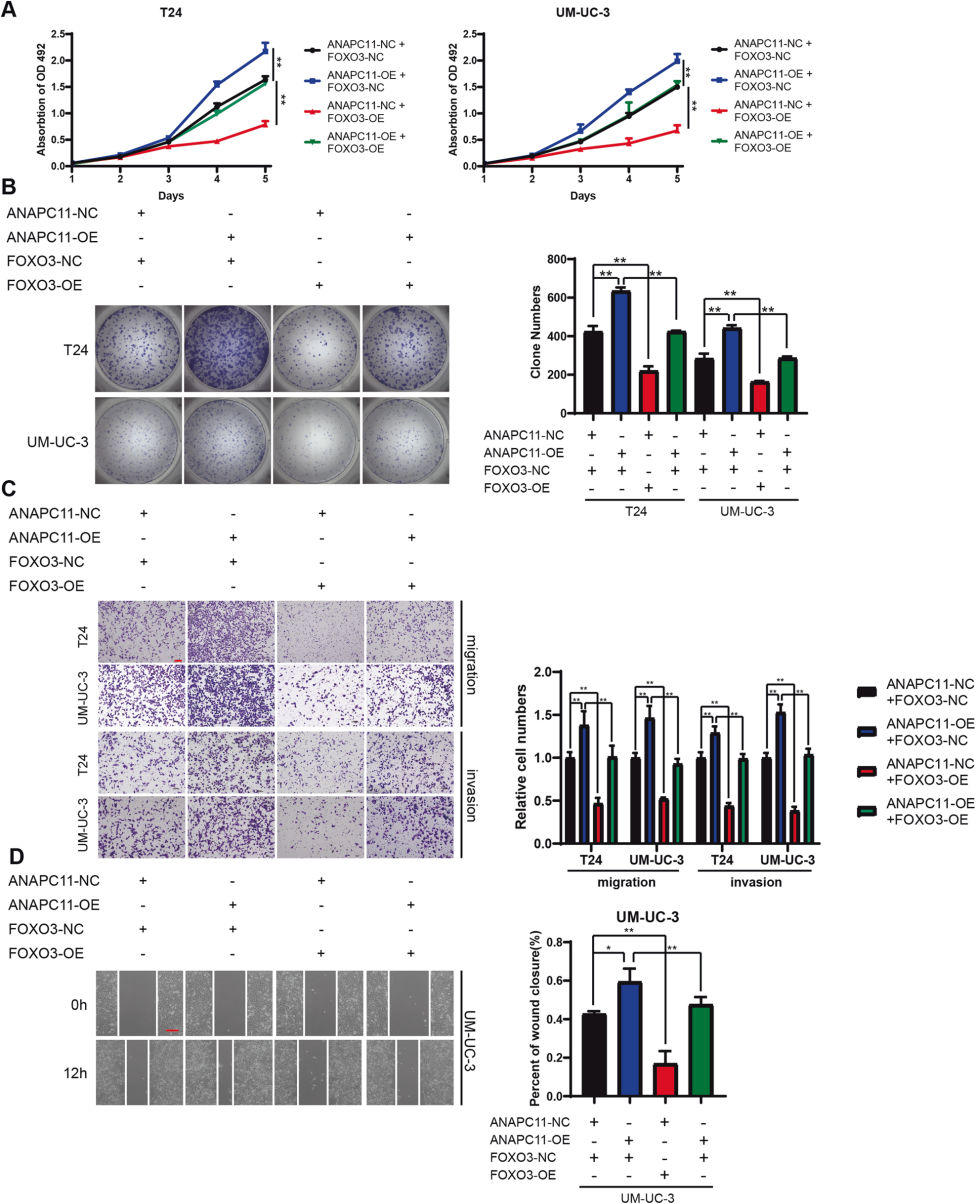
Overexpression of FOXO3 abrogates ANAPC11-mediated progression in UBC cells. **A**–**D** ANAPC11-NC or -OE UBC cells were transiently transfected with pcDNA3.1 plasmids containing an NC sequence or the FOXO3 ORF. The growth rates of cells were evaluated by MTS assays (**A**); the colony formation abilities were measured by colony formation assays (**B**); the migration and invasion of cells were quantified by Transwell migration and invasion assays (**C**); and the percent of wound healing was evaluated via wound healing assays (**D**). Data are shown as mean ± SD. ANOVA, ^*^P < 0.05, ^**^P < 0.01. Scale bar = 100 μm (**C**). Scale bar = 200 μm (**D**).

Based on the data above, we regarded FOXO3 as a downstream effector of ANAPC11 that regulates the proliferation and invasiveness of UBC cells. To provide more supportive evidence, we transfected the pcDNA3.1 vector along with plasmids containing an NC sequence or the FOXO3 ORF into ANAPC11-NC or OE cells. We observed that the upregulation of FOXO3 inhibited the proliferation of ANAPC11-NC cells, as quantified by MTS and colony formation assays (Fig. 5A, B). NC T24 and UM-UC-3 cells with FOXO3 overexpression had weaker migration and invasion capacities than the corresponding control cells (Fig. 5C, D). Supplementation of FOXO3 into T24 and UM-UC-3 cells successfully reversed the oncogenic effects of ANAPC11 overexpression (Fig. 5A, B). Moreover, the elevation of the FOXO3 level abolished the pro-metastasis effects of ANAPC11 (Fig. 5C, D). These experiments suggested that ANAPC11 promoted UBC cell growth and metastasis through the downregulation of FOXO3.

### FOXO3 promotes the transcription of the cyclin-dependent kinase (CDK) inhibitor p21 and GULP1

**Fig. 6:**
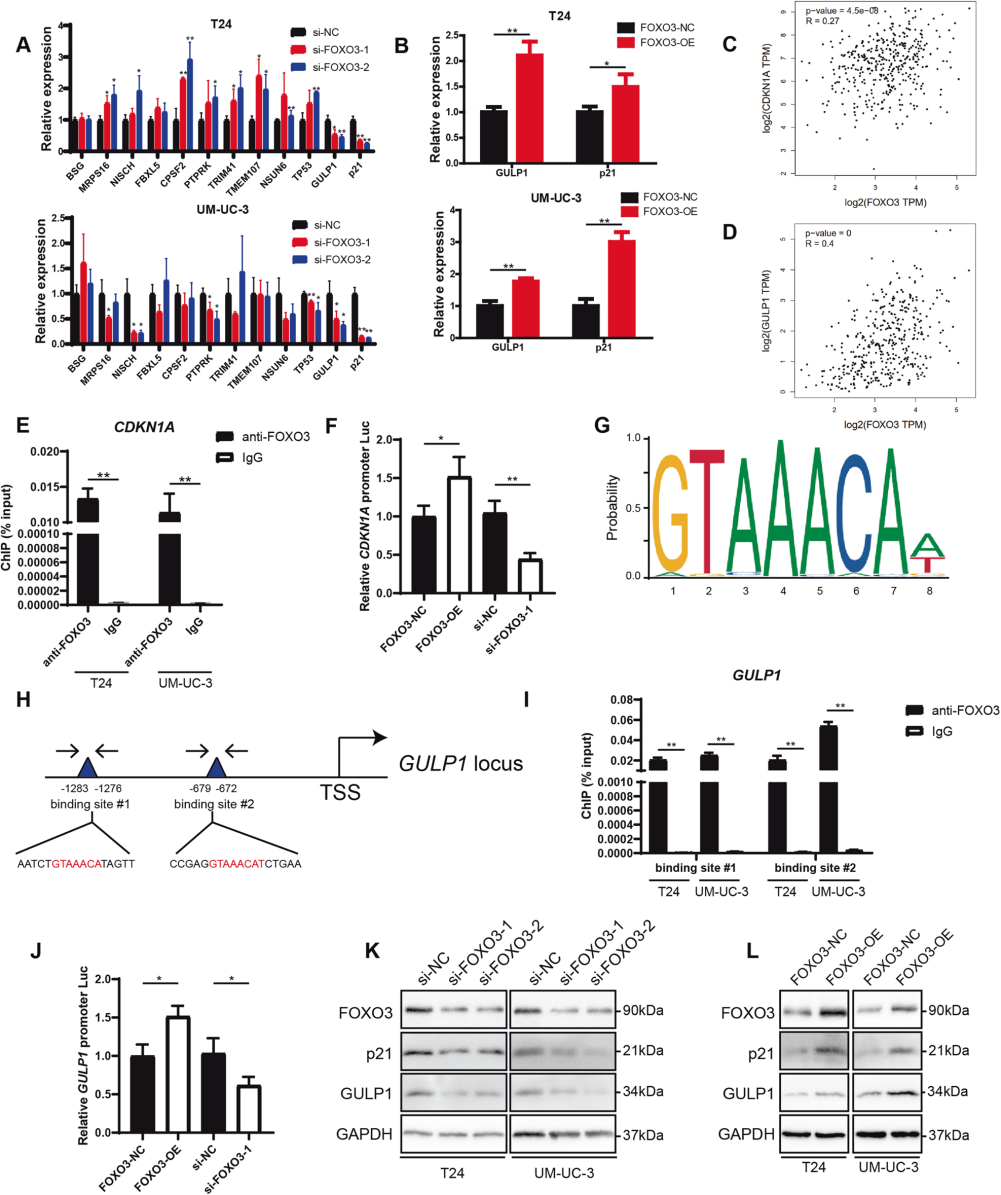
FOXO3 promotes the transcription of the CDK inhibitor p21 and GULP1. **A** The mRNA expression of 12 potential FOXO3 downstream effectors when FOXO3 was silenced in UBC cells, measured by RT–PCR. **B** The mRNA expression of GULP1 and p21 in T24 and UM-UC-3 cells overexpressing FOXO3, measured by RT-PCR. **C**, **D** Correlation analysis of CDKN1A and FOXO3 mRNA (**C**) and correlation analysis of GULP1 and FOXO3 mRNA (**D**) in TCGA-BLCA. **E** Enrichment of the *CDKN1A* promoter with anti-FOXO3 or IgG measured by ChIP followed by RT-PCR. **F** Luciferase reporter assays in 293 T to evaluate the regulation of FOXO3 expression on *CDKN1A* transcriptional activity. Firefly luciferase activity was normalized to Rluc intensity. **G** The prediction of FOXO3 binding motif via JASPAR. **H** Illustration of two potential binding sites of FOXO3 in *GULP1* promoter. **I** Enrichment of two binding sites in *GULP1* promoter with anti-FOXO3 or IgG measured by ChIP followed by RT-PCR. **J** The effect of FOXO3 expression on *GULP1* transcriptional activity, evaluated by dual luciferase reporter assays. **K**, **L** Western blot analysis showed the expression of FOXO3, p21, and GULP1 when the FOXO3 level was downregulated (**K**) or upregulated (**L**) in UBC cells. Data are shown as mean ± SD. Unpaired, two-tailed student’s *t*-test, ^*^P < 0.05, ^**^P < 0.01.

FOXO3 has been reported to influence cell proliferation, apoptosis, and invasiveness in a series of malignancies, but the specific regulatory mechanisms have yet to be fully elucidated [43]. Since ANAPC11 destabilized FOXO3 by ubiquitination and FOXO3 was recognized as a transcription factor, we hypothesized that FOXO3 might regulate the transcription of a series of key genes involved in urothelial bladder carcinogenesis. Therefore, we predicted the FOXO3 binding motifs in genomic DNA by utilizing bioinformatic tools, including GRNdb and Cistrome Data Browser. Then, we conducted RT-PCR to determine whether the expression of candidate genes was consistently altered when FOXO3 was silenced. In total, we analyzed 12 genes in UBC cells with FOXO3 knockdown and found that the mRNA levels of the CDK inhibitor p21 and GULP1 were consistently decreased (Fig. 6A). When FOXO3 was overexpressed, p21 and GULP1 mRNA expression was elevated (Fig. 6B). Correlation analysis was conducted with the Gepia server, and the mRNA levels of both p21 and GULP1 exhibited a positive correlation with the FOXO3 mRNA level in UBC specimens (Fig. 6C, D). Therefore, we focused on p21 and GULP1 for further study.

The CDK inhibitor p21 is encoded by *CDKN1A* and is a vital mediator of different cellular processes. The gain of function of p21 is regarded to have robust suppressive effects on tumorigenesis. Ludger Hauck et al. reported that FOXO3 could interact with the Smad-binding region (SBR) in the *CDKN1A* promoter to induce p21 transcription in cardiac myocytes [44]. To test this effect in UBC cells, ChIP was performed. Primers specifically targeting the SBR in the *CDKN1A* promoter successfully amplified sequences in the product obtained with anti-FOXO3 antibodies but not that obtained with IgG (Fig. 6E). Moreover, luciferase reporter assays showed that FOXO3 expression enhanced the transcriptional activity of p21 (Fig. 6F).

GULP1 has been reported to negatively regulate the proliferation and invasion of UBC cells and is frequently silenced in invasive UBC tissues [45]. We used JASPAR to predict the most likely motif for FOXO3 binding (Fig. 6G) and identified two potential binding regions within the promoter of *GULP1*, located in the regions encompassing bp −1283 to −1276 and bp −679 to −672 upstream of the TSS (Fig. 6H). Specific pairs of primers were designed and synthesized for targeting the two regions in ChIP assays. The results revealed that FOXO3 could bind to both regions (Fig. 6I). Moreover, dual-luciferase reporter assays suggested that ectopic FOXO3 expression elevated the transcription level of GULP1 (Fig. 6J).

We further tested the regulatory roles of FOXO3 at the translational level. As expected, silencing FOXO3 decreased the abundances of p21 protein and GULP1 protein, while upregulation of FOXO3 increased the abundances of these proteins (Fig. 6K-L). These results indicated that FOXO3 promoted the transcription of p21 and GULP1.

### Knockout of ANAPC11 inhibits the proliferation and lymph node metastasis of UBC in vivo

**Fig. 7:**
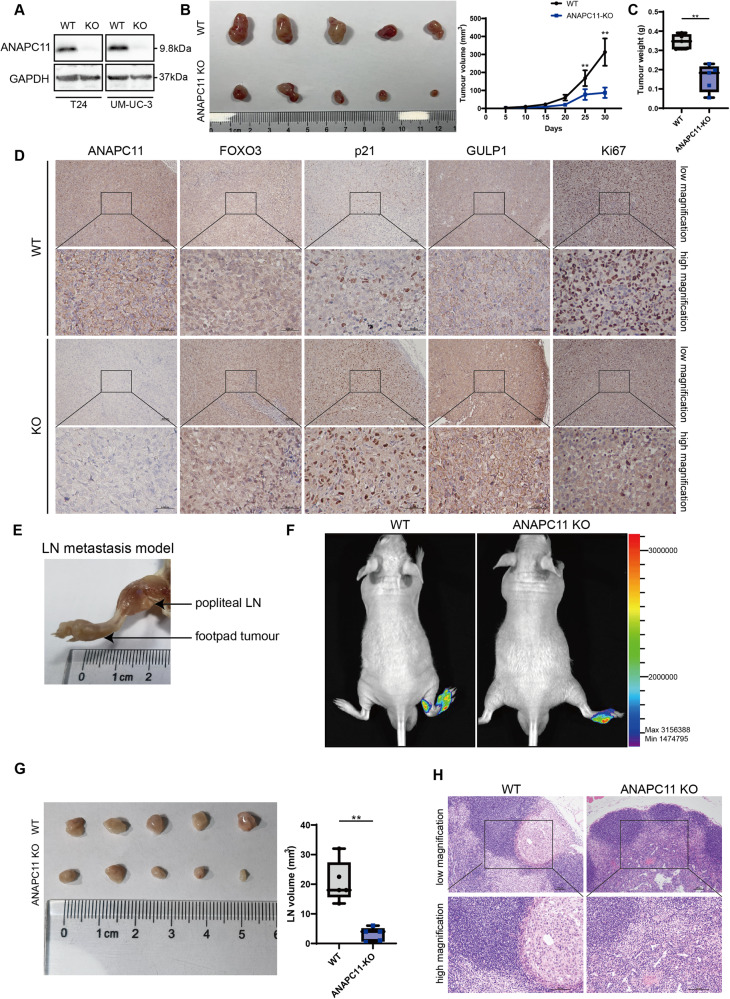
Knockout of ANAPC11 inhibits the proliferation and lymph node metastasis of UBC in vivo. **A** Expression of ANAPC11 and GAPDH in UBC cells infected with CRISPR–Cas9 lentivirus was detected by western blot analysis. **B**, **C** Equal amounts of T24 NC or KO cells were subcutaneously injected into the left flanks of BALB/c nude mice, and tumor volumes (**B**) and weights (**C**) were measured 30 days after injection. ANOVA (**B**) or Mann–Whitney *U* test (**C**). **D** IHC staining detected the expression of ANAPC11, FOXO3, p21, GULP1, and Ki67 in subcutaneous tumors. Scale bar = 100 μm for low magnification and 500 μm for high magnification. (**E)** A representative image of in vivo footpad model. **F** Representative images of both NC and KO groups in the LN metastasis model, whose bioluminescence was detected by an in vivo bioluminescence imaging system. **G** Popliteal LNs were enucleated, and the volumes were measured. Mann–Whitney *U* test. **H** IHC analysis of popliteal LNs. Scale bar = 100 μm. Data are shown as mean ± SD (**B**, **G**) or min to max (**C**). ^**^P < 0.01.

To evaluate the anti-tumor effect of ANAPC11 silencing in vivo, an ANAPC11-KO T24 cell line was established via CRISPR‒Cas9 lentivirus infection. The efficiency of the knockout was checked by western blot analysis (Fig. 7A). Equal amounts of T24 NC and KO cells were subcutaneously injected into the left flanks of BALB/c nude mice, and the tumor volumes and weights in the KO group were significantly decreased compared with those in the NC group (Fig. 7B, C). The expression of ANAPC11 was detected in tumor sections by IHC staining, and knockout of ANAPC11 was found to induce increased expression of FOXO3, p21, and GULP1 and decreased expression of Ki67, a well-known marker for cell proliferation (Fig. 7D). For the LN metastasis model, NC, and KO T24 cells were infected with Lenti-luciferase P2A-Neo lentivirus to express luciferase and selected with G-418. NC or KO cells were injected into the footpads of nude mice, and metastasis from the primary site to the popliteal LNs was observed by an in vivo imaging system (Fig. 7E, F). The invasiveness of UBC cells was reduced in the KO group, and the volume of popliteal LNs was lower (Fig. 7G, H). Collectively, these findings indicated that knockout of ANAPC11 inhibited the proliferation and LN metastasis of UBC cells in vivo.

## Discussion

The aggressiveness of UBC is characterized by muscle invasion, LN metastasis [46, 47], and specific molecular subtypes [5, 48], and involves multiple signaling pathways such as the PI3K/AKT/mTOR [49] and Hippo signaling pathways [50]. In the present study, we revealed a novel mechanism by which the APC/C catalytic subunit ANAPC11 promotes urothelial carcinoma cell proliferation and LN metastasis. Our in vivo and in vitro experiments confirmed that ANAPC11 could mediate the ubiquitination of the Forkhead transcription factor FOXO3 and induce its degradation through the UPS. Furthermore, we confirmed that the expression of the downstream effectors p21 and GULP1 was controlled by the ANAPC11–FOXO3 regulatory axis. These data emphasize the oncogenic roles of ANAPC11 and its clinical value as a potential therapeutic target in UBC.

ANAPC11, also called APC11, is a core catalytic subunit of APC/C [51, 52]. APC/C is a nineteen-subunit cullin-RING E3 Ub ligase that binds with different cofactors to regulate the separation of sister chromatids during the progression from metaphase to anaphase [40, 53]. It degrades a series of targets, including cyclin B, securin, and other D box-containing proteins, via the 26S proteasome [54, 55]. Although high-resolution structural studies using cryo-electron microscopy have advanced our understanding of APC/C, the functions of its subunits/subcomplexes in cancer cells are incompletely elucidated. In one of the previous studies, Youenn Droutt et al. demonstrated that ANAPC11 expression was elevated in colorectal cancer, and upregulated expression implies chromosomal instability, lymphovascular invasion, and residual disease [56]. In our study, we identified FOXO3 as a downstream effector of ANAPC11. First, the physical interaction between ANAPC11 and FOXO3 was confirmed by IP, mass spectrometry, and a GST pulldown assay. Then, we found that the abundance of ANAPC11 negatively regulated the FOXO3 protein level without similarly affecting the FOXO3 mRNA level, suggesting that the regulation was achieved post-transcriptionally. Moreover, CHX assays indicated that ANAPC11 influenced the FOXO3 protein abundance by altering stability. Further MG132 assays proved that this regulation was UPS-dependent. Finally, through IP analysis of FOXO3 ubiquitination, we confirmed that ANAPC11 induced FOXO3 protein degradation through the UPS.

Forkhead box class O (FOXO) proteins constitute a family of evolutionarily conserved transcription factors featuring Forkhead winged helix-turn-helix DNA-binding domains [57, 58]. In humans, there are four members of the FOXO family, namely FOXO1, FOXO3 (also called FOXO3a or FKHRL1), FOXO4, and FOXO6 [59]. All these members bind to a core consensus DNA motif called the Forkhead Response Element (FHRE; 5’-GTAAA(T/C)A-3’ or 5’-TG/ATTTAC-3’) to modulate the transcription of target genes [60]. FOXO3 has been reported to be a longevity-related gene in humans and an important regulator correlated with cancer drug resistance, lymphomagenesis, and Sirtuin signaling [61–64]. In tumors, FOXO proteins generally play a role as tumor suppressors they act as major downstream effectors of the PI3K/AKT pathway, which is thought to be the most frequently activated signaling cascade in human cancers [65–67]. FOXO3 is closely related to autophagy, a process in which misfolded proteins or damaged organelles are degraded in double-membrane vesicles called autophagosomes to maintain redox homeostasis within cells [68, 69]. In UBC, the expression of FOXO3 mRNA in UBC specimens was lower than that in NATs, and a lower FOXO3-positive rate was associated with LN metastasis and a high tumor grade [70]. Moreover, the ratio of phosphorylated FOXO3 (p-FOXO3) to total FOXO3 is related to tumor grade, and in low-grade UBC, a decreased p-FOXO3/FOXO3 ratio suggests a lower recurrence risk [71]. In this study, we confirmed that FOXO3 was a tumor suppressor in UBC. Overexpression of FOXO3 in UBC cells abrogated tumor cell proliferation and migration.

The ANAPC11–FOXO3 regulatory mechanism in tumors could be sophisticated. Previous studies have shown that ANAPC11 could link Ub to the substrate independent of APC/C via the E1 and UbcH5 enzymes, which do not require a D box in the substrate in vitro [39, 72] In addition, deregulation of FOXO3 by aberrant PTM is observed in most malignancies and multiple types of PTMs including phosphorylation, acetylation, ubiquitination and methylation alter the degradation, subcellular localization and DNA-binding affinity of FOXO3 [73–75]. Therefore, the detailed regulatory mechanism of ANAPC11–FOXO3 mediated directly by the classical APC/C and/or through uncharacterized complexes warrants further investigation.

Regarding the FOXO3 downstream targets, we verified that the CDK inhibitor p21 and the engulfment adapter GULP1 are key genes. p21 has been well documented as a tumor suppressor gene, while GULP1 might function as an adapter protein for efficient phagocytosis of apoptotic cells [76]. GULP1 could result in the rearrangement of the actin cytoskeleton via the MAPK pathway, and GULP1 expression positively regulates TGF-β signaling, leading to the inhibition of tumor cell growth [77, 78]. In this study, we confirmed that FOXO3 is bound to the promoter region of *GULP1* to increase its transcriptional activity. We identified two FOXO3 binding sites in the *GULP1* promoter region, whose sequences were identical to the FHRE sequence, the most classical binding motif for FOXO family members.

In addition, the FOXO3–GULP1 axis might contribute to UBC cell metastasis through an incompletely elucidated mechanism. Previous studies have shown that GULP1 is related to the internalization and endosomal trafficking of ligands for lipoprotein receptors [79]. Overexpression of GULP1 caused glycosphingolipid and free cholesterol accumulation in the late endosome/lysosome compartment in cells, while knockdown of GULP1 expression promoted cholesterol flux and enhanced clearance of glycosphingolipids and cholesterol from late endosomes [79]. Dysregulated cholesterol homeostasis results in resistance to ferroptosis and increased cancer metastasis [80]. Indeed, abnormal cholesterol and lipid metabolism in tumor cells might facilitate LN metastasis, as observed for enhanced fatty acid synthase expression in cervical cancer [81] and the adaptative switch of lipid metabolism to fatty acid oxidation, which has been found to be required for LN metastasis [82]. Therefore, future studies are necessary to elucidate the mechanisms by which FOXO3-GULP1 axis-mediated cholesterol, and lipid metabolism promotes LN metastasis in UBC.

**Fig. 8:**
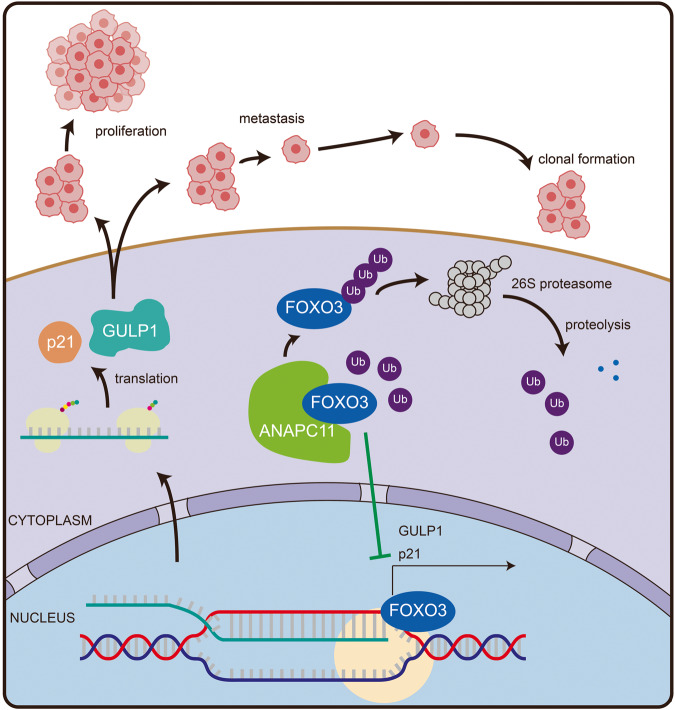
Schematic model of the mechanism underlying the role of ANAPC11 in UBC. Model, we propose that high expression of ANAPC11 degrades FOXO3 through the UPS. FOXO3 can promote the growth and metastasis of UBC by elevating the transcriptional activity of p21 and GULP1.

In conclusion, we demonstrated that ANAPC11 was upregulated in UBC tissues and that high expression of ANAPC11 was associated with poor prognosis in patients. Silencing of ANAPC11 inhibited the growth and invasion of UBC cells in vitro and in vivo. Mechanistically, ANAPC11 degraded FOXO3 through the UPS, and FOXO3 bound to the promoters of *CDKN1A* and *GULP1* to enhance their transcription (Fig. 8). Thus, the ANAPC11–FOXO3 axis might be an important therapeutic target for UBC.

## Supplementary information

Supplementary Figure 1

Supplementary Figure 1 legend

Supplementary Figure 2

Supplementary Figure 2 legend

Supplementary Table 1

Supplementary Table 2

Supplementary Table 3

full length uncropped original western blots

aj-checklist

## Data Availability

Source data are provided with supplementary files at https://depmap.org/portal/ and http://gepia.cancer-pku.cn/. The data of this study can be obtained from the corresponding author upon reasonable request. There are no restrictions on data availability in the current work.
