# Supplementary figures and images for: The APC/C E3 ligase subunit ANAPC11 mediates FOXO3 protein degradation to promote cell proliferation and lymph node metastasis in urothelial bladder cancer
Source: Cell Death Dis. 2023 Aug 12;14(8):516. doi: 10.1038/s41419-023-06000-x (PMC10423259; doi:10.1038/s41419-023-06000-x)

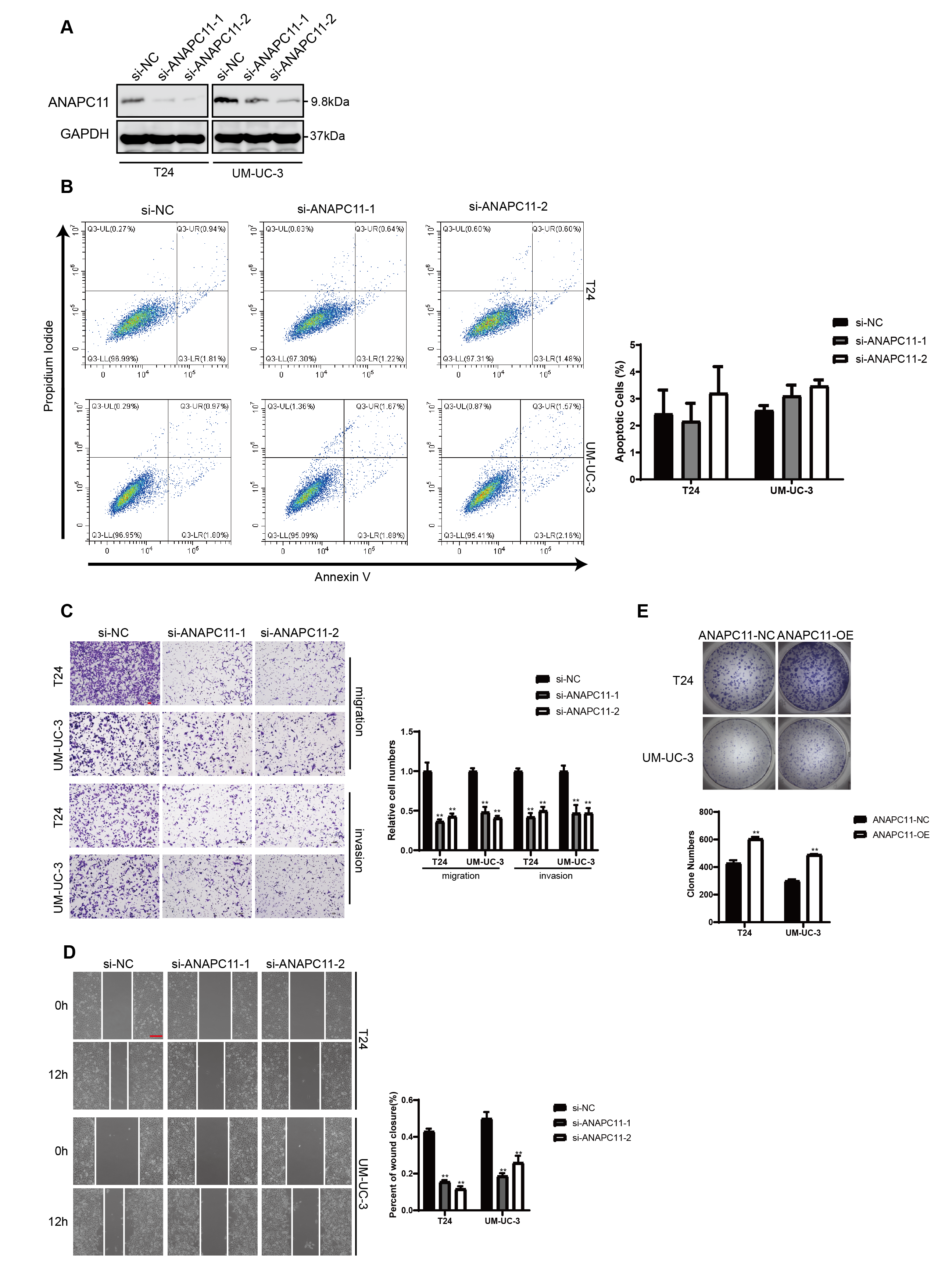

Supplement: Supplementary file 1 — Supplementary Figure 1 [file 41419_2023_6000_MOESM1_ESM.png]

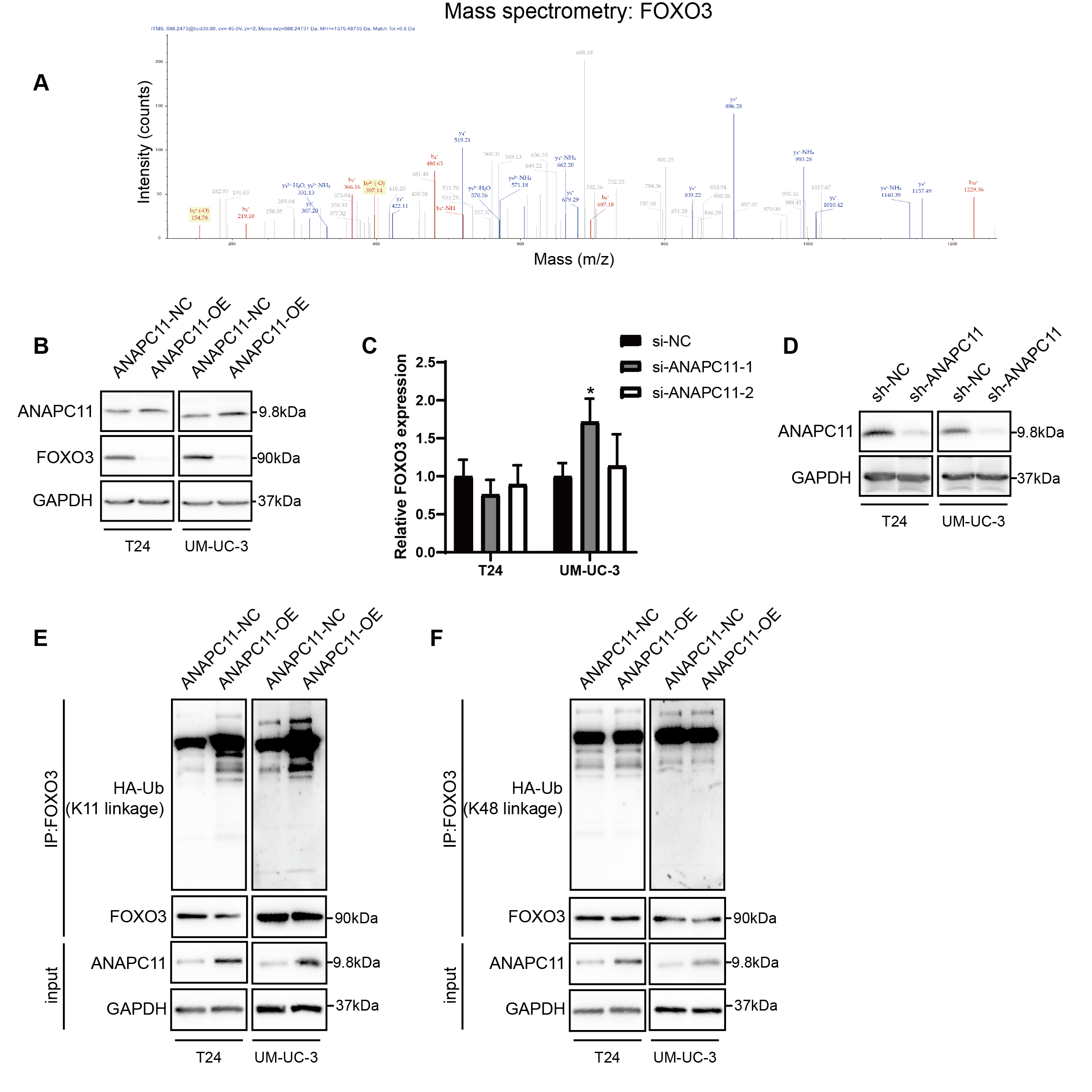

Supplement: Supplementary file 3 — Supplementary Figure 2 [file 41419_2023_6000_MOESM3_ESM.png]
