# Supplementary material for: The APC/C E3 ligase subunit ANAPC11 mediates FOXO3 protein degradation to promote cell proliferation and lymph node metastasis in urothelial bladder cancer
Source: Cell Death Dis. 2023 Aug 12;14(8):516. doi: 10.1038/s41419-023-06000-x (PMC10423259; doi:10.1038/s41419-023-06000-x)
Supplement: Supplementary file 2 — Supplementary Figure 1 legend [file 41419_2023_6000_MOESM2_ESM.docx]

**Supplementary Fig. 1 ANAPC11 knockdown has little impact on apoptosis and inhibits migration of UBC cells in vitro.**

**(A**) Western blot analysis showed the abundance of ANAPC11 in T24 and UM-UC-3 cells treated with indicated siRNAs. (**B**) Representative images and percentage of cell apoptosis. (**C**) Representative images and quantification of UBC cells’ Transwell migration and invasion assays. Unpaired, two-tailed student’s *t* test, Scale bar = 100 μm. (**D**) UBC cells’ migratory abilities evaluated by wound healing assays. Unpaired, two-tailed student’s *t* test, Scale bar = 200 μm. (**E**) Representative images and histogram analysis of colony formation assays with UBC cells. Unpaired, two-tailed student’s *t* test. Data are shown as mean ± SD. ^**^*P*<0.01.
