# Supplementary material for: The APC/C E3 ligase subunit ANAPC11 mediates FOXO3 protein degradation to promote cell proliferation and lymph node metastasis in urothelial bladder cancer
Source: Cell Death Dis. 2023 Aug 12;14(8):516. doi: 10.1038/s41419-023-06000-x (PMC10423259; doi:10.1038/s41419-023-06000-x)
Supplement: Supplementary file 4 — Supplementary Figure 2 legend [file 41419_2023_6000_MOESM4_ESM.docx]

**Supplementary Fig. 2 ANAPC11 regulates the expression of FOXO3.**

**(A**) Mass spectrometry of IP with anti-ANAPC11 antibody. (**B**) The expression of ANAPC11, FOXO3 and GAPDH measured by western blot analysis. (**C**) Relative FOXO3 mRNA expression in UBC cells transfected indicated siRNAs, measured by RT–PCR. (**D**) Western blot analysis showed the silencing efficiency mediated by shRNA. (**E, F**) Western blot analysis followed by IP with anti-HA antibody in T24 and UM-UC-3 cells transfected with K11 linkage HA-Ub plasmid (**E**) or K48 linkage HA-Ub plasmid (**F**). Data are shown as mean ± SD. Unpaired, two-tailed student’s *t* test, ^*^*P*<0.05.
