# Supplementary material for: The APC/C E3 ligase subunit ANAPC11 mediates FOXO3 protein degradation to promote cell proliferation and lymph node metastasis in urothelial bladder cancer
Source: Cell Death Dis. 2023 Aug 12;14(8):516. doi: 10.1038/s41419-023-06000-x (PMC10423259; doi:10.1038/s41419-023-06000-x)
Supplement: Supplementary file 5 — Supplementary Table 1 [file 41419_2023_6000_MOESM5_ESM.docx]

**Supplementary Table 1.**

The oligonucleotides, shRNA, and sgRNA used in this study.

|  | **Sequence (5’-3’)** |
| --- | --- |
| **Oligonucleotides** | |
| si-NC sense | UUCUCCGAACGUGUCACGUTT |
| si-NC antisense | ACGUGACACGUUCGGAGAATT |
| si-ANAPC11-1 sense | GCAUCUGCAGGAUGGCAUUTT |
| si-ANAPC11-1 antisense | AAUGCCAUCCUGCAGAUGCTT |
| si-ANAPC11-2 sense | GCCCUUGAUCAAGAGACCATT |
| si-ANAPC11-2 antisense | UGGUCUCUUGAUCAAGGGCTT |
| si-FOXO3-1 sense | UAGAAUUGGUGCGUGAACGGAAGUC |
| si-FOXO3-1 antisense | GACUUCCGUUCACGCACCAAUUCUA |
| si-FOXO3-2 sense | UAUACGGGAAGCUAGAGCUCCGCUG |
| si-FOXO3-2 antisense | CAGCGGAGCUCUAGCUUCCCGUAUA |
| s**hRNA** | |
| sh-NC | CCTAAGGTTAAGTCGCCCTCGTTCAAGAGACGAGG  GCGACTTAACCTTAGGTTTTTT |
| sh-ANAPC11 | GAAAGCATTTCTAGGTGTTTTCAAGAGAAACACCTA  GAAATGCTTTCTTTTTT |
| **sgRNA** | |
| ANAPC11 KO | CGACGACTGCCCGCTGGTGT |
