# Supplementary material for: The APC/C E3 ligase subunit ANAPC11 mediates FOXO3 protein degradation to promote cell proliferation and lymph node metastasis in urothelial bladder cancer
Source: Cell Death Dis. 2023 Aug 12;14(8):516. doi: 10.1038/s41419-023-06000-x (PMC10423259; doi:10.1038/s41419-023-06000-x)
Supplement: Supplementary file 6 — Supplementary Table 2 [file 41419_2023_6000_MOESM6_ESM.docx]

**Supplementary Table 2.**

The primers used in this study.

|  | **Sequence (5’-3’)** |
| --- | --- |
| **Primers** | |
| FOXO3 Forward | TCTACGAGTGGATGGTGCGTTG |
| FOXO3 Reverse | CTCTTGCCAGTTCCCTCATTCTG |
| BSG Forward | GGCTGTGAAGTCGTCAGAACAC |
| BSG Reverse | ACCTGCTCTCGGAGCCGTTCA |
| NISCH Forward | ACGCTTGACCTGAGCCACAACA |
| NISCH Reverse | CCAGCAATCCATTGTGACTCAGG |
| CPSF2 Forward | CCAAGGCTGTCAGACTTCAAGC |
| CPSF2 Reverse | GCAGCCTTCTAATCCAATGCGTC |
| TRIM41 Forward | CCGAGAATCCAGGAGCCACAAA |
| TRIM41 Reverse | TCCAGGTGCTTCCTCAGTGGTT |
| TMEM107 Forward | CCGAGGAGTATGACAAGCAGGA |
| TMEM107 Reverse | AGATGAGGCTCTGGGTGCTGTT |
| TP53 Forward | CCTCAGCATCTTATCCGAGTGG |
| TP53 Reverse | TGGATGGTGGTACAGTCAGAGC |
| NSUN6 Forward | GATATGGTGGAGGACACAGAAGG |
| NSUN6 Reverse | GATGCCACTTCCTTCACAGACC |
| MRPS16 Forward | GTTGCCCTCAACCTAGACAGGA |
| MRPS16 Reverse | CCGTTTCCTTCGCAGTCTCTCA |
| FBXL5 Forward | GGCAGATTTTAGAGCTTTGTCCTA |
| FBXL5 Reverse | CGAAGACTCTGGCAGCAACCAA |
| PTPRK Forward | CACAGCCATCAATGTCACCACC |
| PTPRK Reverse | CACCTTTGGCTTGTGCTGGTCT |
| GAPDH Forward | CAAGGCTGAGAACGGGAAG |
| GAPDH Reverse | TGAAGACGCCAGTGGACTC |
| GULP1 Forward | ACTGTGGAGCAGCAGATTTCCC |
| GULP1 Reverse | GTCAGCACCTACTGTCTAACGG |
| CDKN1A Forward | AGGTGGACCTGGAGACTCTCAG |
| CDKN1A Reverse | TCCTCTTGGAGAAGATCAGCCG |
| GULP1 promoter #1 Forward | CATGAGATTTGGGGAAAGTCAC |
| GULP1 promoter #1 Reverse | AAAAAGGCATCATTTCACAATTT |
| GULP1 promoter #2 Forward | AATGGGCTGTTGTGTTTTCTCT |
| GULP1 promoter #2 Reverse | CCCCCAGATCATACTCATTTGT |
| CDKN1A promoter Forward | CCTGAGGAGAGCCAACTGCCTGC |
| CDKN1A promoter Reverse | GGAGGACTCCTCTCCTGTGGTGG |
