# Supplementary material for: The APC/C E3 ligase subunit ANAPC11 mediates FOXO3 protein degradation to promote cell proliferation and lymph node metastasis in urothelial bladder cancer
Source: Cell Death Dis. 2023 Aug 12;14(8):516. doi: 10.1038/s41419-023-06000-x (PMC10423259; doi:10.1038/s41419-023-06000-x)
Supplement: Supplementary file 7 — Supplementary Table 3 [file 41419_2023_6000_MOESM7_ESM.docx]

**Supplementary Table 3.**

The E1, E2, E3 and DUB concluded in this study.

| **E1** |  |  |  |  |
| --- | --- | --- | --- | --- |
| UBE1 | UBA6 |  |  |  |
| **E2** |  |  |  |  |
| Ube2A | Ube2B | Ube2C | Ube2D1 | Ube2D2 |
| Ube2D3 | Ube2D4 | Ube2E1 | Ube2E2 | Ube2E3 |
| Ube2G1 | Ube2G2 | Ube2H | Ube2J1 | Ube2J2 |
| Ube2K | Ube2L3 | Ube2N | Ube2NL | Ube2O |
| Ube2Q1 | Ube2Q2 | Ube2QL | Ube2R1 | Ube2R2 |
| Ube2S | Ube2T | Ube2U | Ube2V1 | Ube2V2 |
| Ube2W | BIRC6 |  |  |  |
| **E3** |  |  |  |  |
| ABTB1 | FBXO42 | KLHL34 | RNF121 | TRIM35 |
| ABTB2 | FBXO44 | KLHL4 | RNF122 | TRIM36 |
| AMFR | FBXO45 | KLHL5 | RNF123 | TRIM37 |
| ANAPC11 | FBXO46 | KLHL6 | RNF125 | TRIM38 |
| ANKFY1 | FBXO5 | KLHL7 | RNF126 | TRIM39 |
| ANKIB1 | FBXO6 | KLHL8 | RNF128 | TRIM4 |
| ANUBL1 | FBXO7 | KLHL9 | RNF13 | TRIM40 |
| ARIH1 | FBXO8 | LGALS3BP | RNF130 | TRIM41 |
| ARIH2 | FBXO9 | LINCR | RNF133 | TRIM42 |
| ARMC5 | FBXW11 | LNX1 | RNF135 | TRIM43 |
| ASB10 | FBXW2 | LNX2 | RNF138 | TRIM45 |
| ASB11 | FBXW4 | LOC120824 | RNF139 | TRIM46 |
| ASB13 | FBXW5 | LOC123103 | RNF14 | TRIM47 |
| ASB14 | FBXW7 | LOC150739 | RNF141 | TRIM48 |
| ASB15 | FBXW8 | LOC283116 | RNF144 | TRIM49 |
| ASB16 | FLJ30092 | LOC286526 | RNF145 | TRIM5 |
| ASB17 | GAN | LOC339745 | RNF146 | TRIM50 |
| ASB18 | GMCL1 | LOC388419 | RNF148 | TRIM52 |
| ASB2 | GTF2H2 | LOC390231 | RNF149 | TRIM54 |
| ASB3 | GZF1 | LOC399937 | RNF150 | TRIM55 |
| ASB4 | HACE1 | LOC399939 | RNF151 | TRIM56 |
| ASB5 | HECTD1 | LOC441061 | RNF152 | TRIM58 |
| ASB6 | HECTD2 | LOC51136 | RNF157 | TRIM59 |
| ASB7 | HECTD3 | LONRF1 | RNF165 | TRIM6 |
| ASB8 | HECW1 | LONRF2 | RNF166 | TRIM60 |
| ASB9 | HECW2 | LONRF3 | RNF167 | TRIM61 |
| ATRX | HERC1 | LRRC29 | RNF168 | TRIM62 |
| BACH1 | HERC2 | LRSAM1 | RNF169 | TRIM63 |
| BACH2 | HERC3 | LZTR1 | RNF17 | TRIM64 |
| BARD1 | HERC4 | MAP3K1 | RNF170 | TRIM65 |
| BCL6 | HERC5 | MARCH1 | RNF175 | TRIM67 |
| BCL6B | HERC6 | 'MARCH2 | RNF180 | TRIM68 |
| BFAR | HIC1 | 'MARCH3 | RNF181 | TRIM69 |
| BIRC2 | HIC2 | 'MARCH4 | RNF182 | TRIM7 |
| BIRC3 | HLTF | 'MARCH5 | RNF183 | TRIM72 |
| BIRC4 | HUWE1 | 'MARCH6 | RNF185 | TRIM73 |
| BIRC7 | IBRDC1 | 'MARCH7 | RNF186 | TRIM74 |
| BIRC8 | IBRDC2 | 'MARCH8 | RNF187 | TRIM75 |
| BMI1 | IBRDC3 | 'MARCH9 | RNF19 | TRIM8 |
| BRAP | IBTK | MDM2 | RNF190 | TRIM9 |
| BRCA1 | IPP | MDM4 | RNF2 | TRIML1 |
| BTBD1 | ITCH | MGC23270 | RNF20 | TRIP12 |
| BTBD10 | IVNS1ABP | MGRN1 | RNF207 | TTC3 |
| BTBD11 | KBTBD10 | MIB1 | RNF208 | TULP4 |
| BTBD12 | KBTBD11 | MIB2 | RNF212 | UBE3A |
| BTBD14A | KBTBD2 | MID1 | RNF213 | UBE3B |
| BTBD14B | KBTBD3 | MID2 | RNF214 | UBE3C |
| BTBD2 | KBTBD4 | MKRN1 | RNF215 | UBE4A |
| BTBD3 | KBTBD5 | MKRN2 | RNF24 | UBE4B |
| BTBD6 | KBTBD6 | MKRN3 | RNF25 | UBOX5 |
| BTBD7 | KBTBD7 | MNAT1 | RNF26 | UBOX5 |
| BTBD8 | KBTBD8 | MSL2L1 | RNF31 | UBR1 |
| BTBD9 | KCNA1 | MYCBP2 | RNF32 | UBR2 |
| BTRC | KCNA10 | MYLIP | RNF34 | UBR4 |
| C13orf7 | KCNA2 | MYNN | RNF38 | UBR5 |
| C14orf4 | KCNA3 | NEDD4 | RNF39 | UHRF1 |
| C16orf28 | KCNA4 | NEDD4L | RNF4 | UHRF2 |
| C16orf44 | KCNA5 | NEURL | RNF40 | UNK |
| C1orf164 | KCNA6 | NEURL2 | RNF41 | VHL |
| C1orf166 | KCNA7 | NFX1 | RNF43 | VPS11 |
| C8ORFK36 | KCNB1 | NFXL1 | RNF44 | VPS18 |
| CBL | KCNB2 | NHLRC1 | RNF5 | VPS41 |
| CBLB | KCNC1 | NOSIP | RNF6 | VPS8 |
| CBLC | KCNC2 | NSMCE1 | RNF7 | WDSUB1 |
| CBLL1 | KCNC3 | OSTM1 | RNF8 | WHSC1 |
| CCIN | KCNC4 | OTUD7A | RP3-509I19.5 | WSB1 |
| CCNB1IP1 | KCND1 | OTUD7B | RSPRY1 | WSB2 |
| CCNF | KCND2 | PARC | SF3B3 | WWP1 |
| CGRRF1 | KCND3 | PARK2 | SH3RF1 | WWP2 |
| CHFR | KCNG1 | PATZ1 | SH3RF2 | ZBTB1 |
| CISH | KCNG3 | PCGF1 | SHKBP1 | ZBTB10 |
| CNOT4 | KCNRG | PCGF2 | SHPRH | ZBTB11 |
| CPSF1 | KCNS1 | PCGF3 | SIAH1 | ZBTB12 |
| DCST1 | KCNS2 | PCGF5 | SIAH2 | ZBTB16 |
| DDB1 | KCNS3 | PCGF6 | SKP2 | ZBTB17 |
| DPF1 | KCNV1 | PDZRN3 | SMURF1 | ZBTB2 |
| DTX1 | KCTD1 | PDZRN4 | SMURF2 | ZBTB20 |
| DTX2 | KCTD10 | PEX10 | SOCS1 | ZBTB22 |
| DTX3 | KCTD12 | PEX12 | SOCS2 | ZBTB24 |
| DTX3L | KCTD13 | PHF7 | SOCS3 | ZBTB25 |
| DTX4 | KCTD14 | PJA1 | SOCS4 | ZBTB26 |
| DZIP3 | KCTD15 | PJA2 | SOCS5 | ZBTB3 |
| ENC1 | KCTD16 | PML | SOCS6 | ZBTB32 |
| FANCL | KCTD17 | PPIL2 | SOCS7 | ZBTB33 |
| FBXL10 | KCTD2 | PRPF19 | SPOP | ZBTB37 |
| FBXL11 | KCTD20 | PXMP3 | SPRYD5 | ZBTB38 |
| FBXL12 | KCTD21 | RAB40A | SPSB1 | ZBTB39 |
| FBXL13 | KCTD3 | RAB40B | SPSB2 | ZBTB4 |
| FBXL14 | KCTD4 | RAB40C | SPSB3 | ZBTB40 |
| FBXL15 | KCTD5 | RABGEF1 | SPSB4 | ZBTB41 |
| FBXL16 | KCTD6 | RAD18 | STUB1 | ZBTB43 |
| FBXL18 | KCTD7 | RAG1 | SYVN1 | ZBTB44 |
| FBXL2 | KCTD7 | RAPSN | TCEB3 | ZBTB45 |
| FBXL20 | KCTD8 | RBBP6 | TNFAIP1 | ZBTB46 |
| FBXL3 | KCTD9 | RBCK1 | TNFAIP3 | ZBTB48 |
| FBXL4 | KEAP1 | RBX1 | TOPORS | ZBTB5 |
| FBXL5 | KIAA0317 | RC3H1 | TRAF2 | ZBTB6 |
| FBXL6 | KIAA1333 | RC3H2 | TRAF3 | ZBTB7A |
| FBXL7 | KIAA1542 | RCBTB1 | TRAF4 | ZBTB7B |
| FBXL8 | KLHDC5 | RCBTB2 | TRAF5 | ZBTB7C |
| FBXO10 | KLHDC6 | RCHY1 | TRAF6 | ZBTB8 |
| FBXO11 | KLHL1 | RFFL | TRAF7 | ZBTB9 |
| FBXO15 | KLHL10 | RFPL1 | TRAIP | ZFAND3 |
| FBXO16 | KLHL11 | RFPL3 | TRIAD3 | ZFAND5 |
| FBXO17 | KLHL12 | RFPL4A | TRIM10 | ZFAND6 |
| FBXO18 | KLHL13 | RFPL4B | TRIM11 | ZFP161 |
| FBXO2 | KLHL14 | RFWD2 | TRIM13 | ZFPL1 |
| FBXO21 | KLHL15 | RFWD3 | TRIM15 | ZNF131 |
| FBXO22 | KLHL17 | RHOBTB1 | TRIM17 | ZNF179 |
| FBXO24 | KLHL18 | RHOBTB2 | TRIM2 | ZNF238 |
| FBXO25 | KLHL20 | RHOBTB3 | TRIM21 | ZNF294 |
| FBXO27 | KLHL21 | RING1 | TRIM22 | ZNF295 |
| FBXO28 | KLHL22 | RKHD1 | TRIM23 | ZNF313 |
| FBXO3 | KLHL23 | RKHD2 | TRIM24 | ZNF364 |
| FBXO30 | KLHL24 | RKHD3 | TRIM25 | ZNF509 |
| FBXO31 | KLHL25 | RKHD4 | TRIM26 | ZNF598 |
| FBXO32 | KLHL26 | RNF10 | TRIM27 | ZNF645 |
| FBXO33 | KLHL28 | RNF103 | TRIM28 | ZNF650 |
| FBXO34 | KLHL29 | RNF11 | TRIM3 | ZNRF1 |
| FBXO36 | KLHL3 | RNF111 | TRIM31 | ZNRF2 |
| FBXO38 | KLHL30 | RNF113A | TRIM32 | ZNRF3 |
| FBXO4 | KLHL31 | RNF113B | TRIM33 | ZNRF4 |
| FBXO40 | KLHL32 | RNF12 | TRIM34 | ZSWIM2 |
| FBXO41 |  |  |  |  |
| **DUB** |  |  |  |  |
| USP29 | USP32 | USP7 | UCHL3 | USP19 |
| YOD1 | VCPIP1 | COPS6 | USP3 | USP21 |
| MINDY2 | USP43 | USP47 | CYLD | USP2 |
| USP26 | BRCC3 | EIF3F | USP51 | OTUD6B |
| OTUB1 | USP28 | USP34 | USPL1 | OTUD6A |
| ZUP1 | USP45 | PSMD7 | USP27X | USP50 |
| OTUB2 | PSMD14 | USP9Y | PAN2 | OTUD3 |
| MINDY1 | USP16 | EIF3H | USP22 | OTUD5 |
| OTUD7A | MPND | USP9X | USP53 | USP20 |
| MINDY4 | USP30 | ATXN3 | USP42 | USP33 |
| USP5 | MYSM1 | USP24 | USP54 | OTUD1 |
| OTUD7B | USP14 | USP41 | USP10 | USP12 |
| MINDY3 | STAMBP | ATXN3L | USP39 | OTUD4 |
| USP13 | USP40 | JOSD1 | USP11 | USP46 |
| TNFAIP3 | STAMBPL1 | JOSD2 | USP15 | OTUD4P1 |
| USP37 | USP48 | BAP1 | USP4 | OTULIN |
| USP6 | PRPF8 | UCHL5 | USP8 | USP17L2 |
| ZRANB1 | USP31 | UCHL1 | USP36 | OTULINL |
| USP17L24 |  |  |  |  |
