# Supplementary material for: The APC/C E3 ligase subunit ANAPC11 mediates FOXO3 protein degradation to promote cell proliferation and lymph node metastasis in urothelial bladder cancer
Source: Cell Death Dis. 2023 Aug 12;14(8):516. doi: 10.1038/s41419-023-06000-x (PMC10423259; doi:10.1038/s41419-023-06000-x)

Fig. 1B

ANAPC11

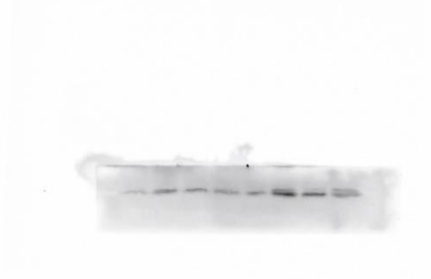

GAPDH

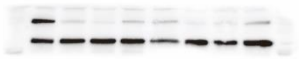

Fig. 1C

ANAPC11

GAPDH

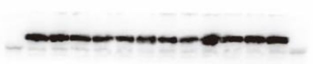

Supplementary Fig. 1A

ANAPC11

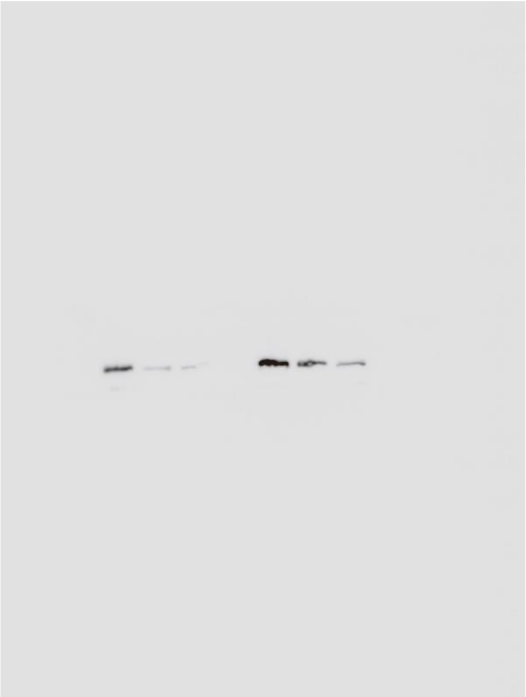

GAPDH

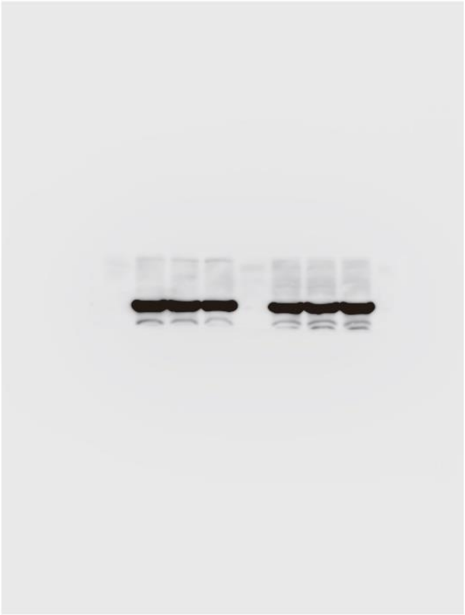

Fig. 2D

ANAPC11

GAPDH

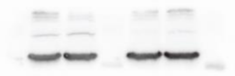

Fig. 7A

ANAPC11

GAPDH

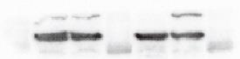

Fig. 3A

FOXO3

ANAPC11

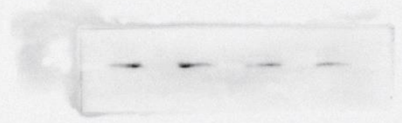

Fig. 3B

IP-His

IP-ANAPC11

input-His

input-GAPDH

Fig. 3C

ANAPC11

FOXO3

GAPDH

Fig. 3D

FOXO3

GST

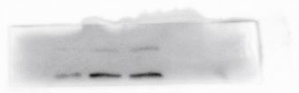

Fig. 3E

T24 FOXO3

T24 GAPDH

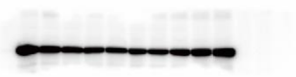

UM-UC-3 FOXO3

UM-UC-3 GAPDH

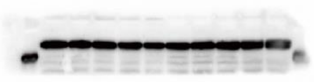

Fig. 3F

ANAPC11

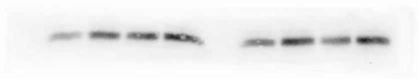

FOXO3

GAPDH

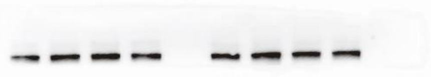

Fig. 3G

IP: FOXO3
Ub T24

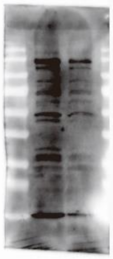

IP: FOXO3
Ub UM-UC-3

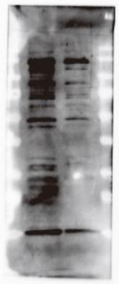

IP: FOXO3
FOXO3

input ANAPC11
T24

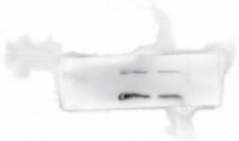

input ANAPC11
UM-UC-3

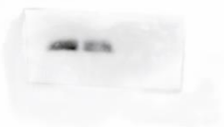

input GAPDH
T24

input GAPDH
UM-UC-3

Fig. 4A

FOXO3

GAPDH

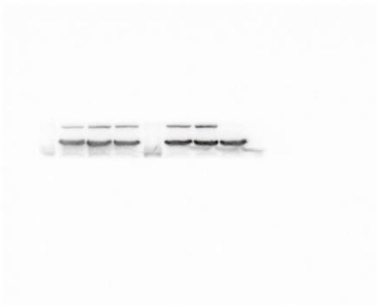

Fig. 6K

FOXO3

p21
same band with different exposure time

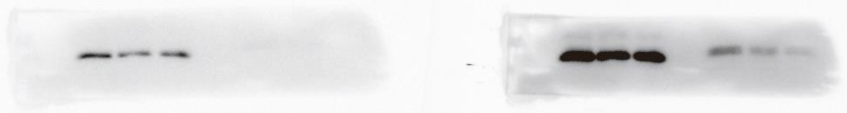

GULP1

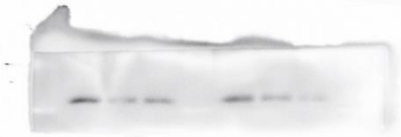

GAPDH

Fig. 6L

FOXO3

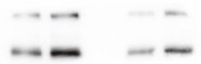

p21

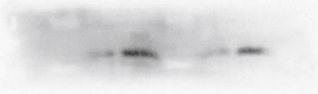

GULP1

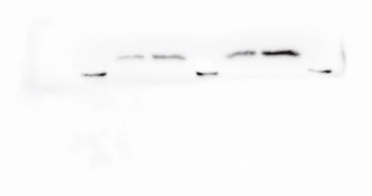

GAPDH

Supplementary Fig. 2B

ANAPC11

FOXO3

GAPDH

Supplementary Fig. 2D

ANAPC11

GAPDH

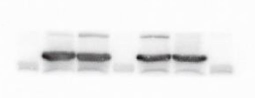

Supplementary Fig. 2E-F

IP:HA

T24

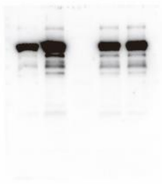

UM-UC-3

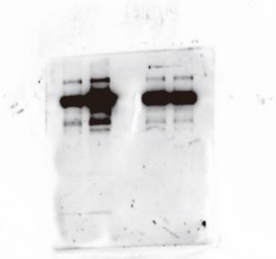

IP:FOXO3

T24

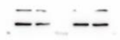

UM-UC-3

input:ANAPC11

T24

UM-UC-3

input:GAPDH

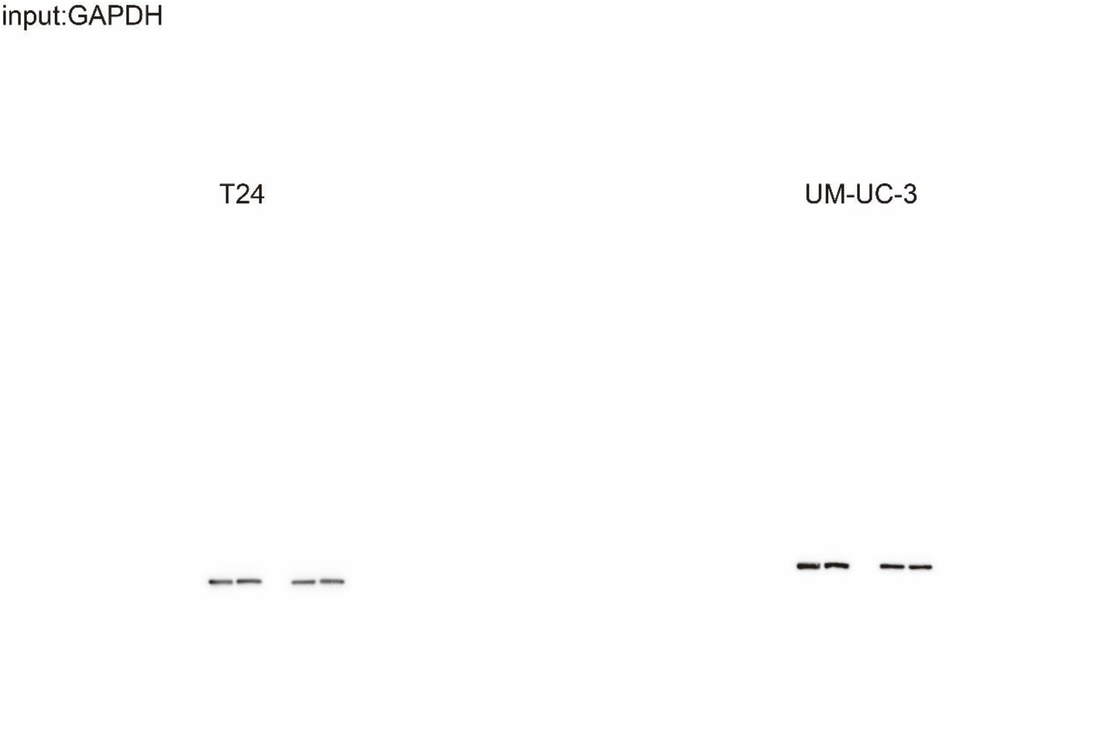

Supplement: Supplementary file 8 — full length uncropped original western blots [file 41419_2023_6000_MOESM8_ESM.pdf]
